# A Case of Strangulated Hernia

**Published:** 1853-03

**Authors:** Charles Fishback

**Affiliations:** Shelbyville, Ind.


ART. III.—*A Case of Strangulated Hernia.* By CHARLES FISHBACK, M. D., of Shelbyville, Ind.

*To the Editors of the Western Journal of Medicine:*

GENTLEMEN: The following case of fatal strangulated hernia has been kindly furnished by my friend and former pupil, Charles Fishback, M. D., now in full and successful practice in Shelbyville, Ind.

It was primarily intended and offered for my private perusal and use, but possessing, as you will perceive, several points of practical interest and value, and in recognition of the trite trueism that "our *fatal* cases are often our most *instructive*, and therefore valuable, ones," I have obtained the consent of Dr. F., and herewith enclose it, with these explanations, for insertion in your journal, if approved by you.

Very respectfully yours,

SAM'L B. RICHARDSON.

J.C., æt. 52. Has been for many years a hotel keeper; has been the subject of oblique inguinal hernia of the right side for twenty years; supposed to have been produced by lifting at a "house-raising." Temperament sanguine-nervous, complexion rather light, with black hair, moderately corpulent. Has been a full liver, and rather free drinker, habitually. His general health has always been good.

When the hernia first appeared, he wore a truss, the form of which is unknown, but for fifteen years omitted it altogether. For three or four years past has worn a heavy leaden truss, of oval form, about two by one and a quarter inches, adjusted by means of straps.

July 27th, 1852, at three o'clock, P. M., he was attacked with colicky pain, more in the hypogastric region than elsewhere. He took some domestic remedies, and also some tincturæ capsici and opii combined, regarding it as colic, from which he frequently suffered. The pain increasing in severity, W. A. Pugh, M. D., of Shelbyville, Indiana, was called to see him at 6, P. M. Found him suffering great pain in the hypogastric and umbilical regions; pulsations sixty-five per minute; tongue clean; skin soft and moist; no thirst. His bowels had not been evacuated for five or six days, as was his ordinary habit. Ordered ℞. — Extract zingiberis ʒss, tincture opii et camphoræ ʒj, tinct. opii gtt. xv, sacchar. albi q. s., to be taken at once, and to be repeated in an hour if the pain continued. Hot pediluvia immediately, and a brisk cathartic at bed time. Four pills of the Compound Colocynth Pills of U. S. D. The anodyne draught was repeated at seven o'clock.

About two o'clock, A. M. 28th, Dr. Pugh was recalled. He had vomited several times, and the family supposed had thrown up the pills. Pain still severe; no change in the other symptoms. Ordered ℞.—Hydrargyri proto-chloridi, ℈ss; pulv. ext. colocynth compd., grs. v; pulv. ipecac compd., ℈ss; mix and take at once. Sinapism to the entire abdomen, and hot pediluvia, which had been neglected.

Seven, A. M.—No change in general symptoms; has vomited but once since three o'clock; partial relief of pain, which has now assumed a paroxysmal form, recurring every ten or fifteen minutes, and is most severe in the epigastric region. Ordered ℞.—Ol. ricini, ℥j; ol. terebinth, ʒss; to be taken at once and repeated every two hours until his bowels are moved.

Nine, A. M.—Vomiting continues, the oil being rejected. Ordered large injections of tepid water, impregnated with salt, which soon emptied the lower bowel; evacuations scybalous and very hard. Ordered a Seidlitz powder, to be repeated in an hour.

Twelve, M—Bowels still unmoved; pulsations seventy-five per minute; tongue slightly coated with white fur. Seidlitz powder also rejected. Vomited matter has been bilious mixed with ingesta, but is now stercoraceous, and very offensive; pain in right groin excessive *during the act of vomiting, but at no other time.* Strict examination (which had been made at every visit after the first) was again made for the hernial tumor, but without being able to detect any, the patient all the time insisting, "*There's nothing wrong there.*" *No tenderness of the inguinal region was manifested by a diligent use of the taxis.* Administered ol. ricini, ℥j., ol. terebinth, ʒss., tinct. opii, ʒj., per anum.

At three o'clock, P. M., Dr. S. D. Day was called in consultation. All the symptoms aggravated; pulsations eighty-five per minute. Ordered Hydrarg. proto-chloridi, ℈j., after which there was no vomiting for two hours.

Conference at six, P. M. Bowels still unopened ; pulsations ninety per minute. Ordered ol. croton tiglii, gtt. ij., ol. ricini, ℥j. Repeat injections, and apply cloths wrung out of hot water to the abdomen.

29th, A. M.—Patient continued to grow worse through the night, during which the ol. croton was twice repeated. Bowels still unmoved ; injections return unchanged ; stomach retains nothing ; vomiting stercoraceous, copious and very offensive ; pulsations one hundred and twenty per minute ; tenderness of epigastrium great, but no pain in the groin, save during the act of vomiting; abdomen tympanitic, except in the right lumbar and iliac regions, which are *emphatically dull*, particularly over the track of the ascending colon.

At seven, A. M., Dr. Fishback was added to the conference. Ordered speedy vesication of the superior portion of the abdomen, which was effected in fifteen minutes with a mixture of aqua ammoniæ., f ol. oliv, tinct. capsici and chloroform. Repeat ol. croton and injections, and also ℥j of ℞. camphora ʒiij, chloroform f. ℥j, vitelli ovi j ; triturate and add aquæ q. s. to make f.℥iv.

Twelve, M. — No special change of symptoms ; pulsations one hundred and forty per minute ; vomiting continues but is less frequent ; the matter dark and excessively offensive. Applied electro-galvanism to the right lumbar region, which excited peristaltic action, with strong desire to stool, but only a *small* portion of fecal matter passed.

Six, P. M. — Patient sinking rapidly, almost pulseless. Applied electro-galvanism again, with similar effect. He continued to grow worse until 2 A. M., July 30th, when he expired.

*Autopsy thirteen hours after death, by Drs. Pugh and Fishback. Dr. Day present.*

The anterior abdominal parietes very thick, there being a thickness of two and a half inches of adipose tissue. The omentum majus was found slightly livid throughout; the small intestine considerably injected, and a portion of it in the right illiac region livid. Passing the hand along between the inner surface of the anterior abdominal wall and the contents of the cavity, in search of adhesions, and to ascertain the condition of the coecum and ascending colon, which were supposed, before death, from the great dulness on percussion of that region, and from the distinctly marked outline of the bowel, as felt by the hand, to contain impacted feces, a protrusion of the small intestine into the right inguinal canal was discovered, and to its particular examination attention was directed, both before and after the extrication of the strangulated intestine.

It was a small knuckle, extending about five-eighths of an inch into the canal, which was nearly horizontal when the body stood upright, the internal ring being almost directly opposite to the external, the entering arm of the knuckle lying above the escaping arm, considering the body in the erect position. The upper arm *only*, and *only the superior border of it*, was strangulated, being compressed by a portion of the tendinous margin of the ring, the *inferior border of the same arm lying loose in the ring*, as did also the entire calibre of the lower arm, wholly free from compression.

The internal ring, and the canal, were about an inch in diameter, the ring feeling hard to the finger, much like the tense margin of the os uteri, during contraction. Its form was as if two portions of its circumference were flattened, so as to form an acute angle, instead of being nearly oval; or as if the superior extremity of the oval were made an acute angle by flattening its margin from that point.

The intestine was free from adhesion to the ring or canal and *the hernial portion entirely empty.* The coecum and ascending colon were partially filled with hardened fæces.

No hernial tumor could be felt after the most careful palpation by Drs. Pugh, Day and Fishback, either before or after death. This may be accounted for *by the smallness of the knuckle, its perfect emptiness*, and the great thickness of the anterior abdominal wall—three inches.

There was *no tenderness at the site of the stricture*, nor nearer than the epigastrium, manifested on careful and repeated taxis and percussion during life.

The autopsy proved Mr. Liston to be in error in saying—"Strangulation arises, not from any change in the neck of the sac, or in the tendinous aperture, but from increase of volume in the protruded parts, caused by accumulation of the solid, fluid, or gaseous contents of the bowel;" for, in this case, *the knuckle was entirely empty*, the portion of it surrounded by the margin of the ring forming the base, and the other doubled extremity of the knuckle the apex of a pyramid, which passed out of the stricture without the slightest difficulty as to room, the upper border *only of but* ONE *arm* of the knuckle being, as it were, pinched by a portion of the tendinous margin of the aperture, and so held, the other arm lying loose and movable, having no mark of constriction on it, while about three-fifths of the circumference of the upper arm was deeply indented from pressure of the tendinous margin, applied in the manner above named, and which diminished the breadth of the bowel at that point fully one-sixth.

The pinched portion of intestine was deeply livid, the lividity diminishing gradually in each direction for several inches. The entire peritoneum was injected, the redness diminishing from the inguinal ring as a centre. *There was no pain of the belly* during life, except of the epigastrium, which was *constant*, and of the right inguinal region *during the act of vomiting only. There was no redness of the abdomen, and no heat;* on the contrary, the entire surface was quite cold.

No further examination was allowed by the fami'y, nor were we permitted to remove the strictured portion of intestine for preservation. His family say, that had the strangulation been detected, and an operation proposed, they know he would not have consented to its performance. They also say that on the afternoon of July 25th — two days before his attack — two of his grandsons, about four or five years old, greatly amused themselves and him by striking his abdomen, as he sat in his chair, with their fists, as hard as their strength allowed, for half an hour.

Whether this, and the strong voluntary contractions of the abdominal muscles to resist each blow, had any effect in producing the strangulation is matter of conjecture.

